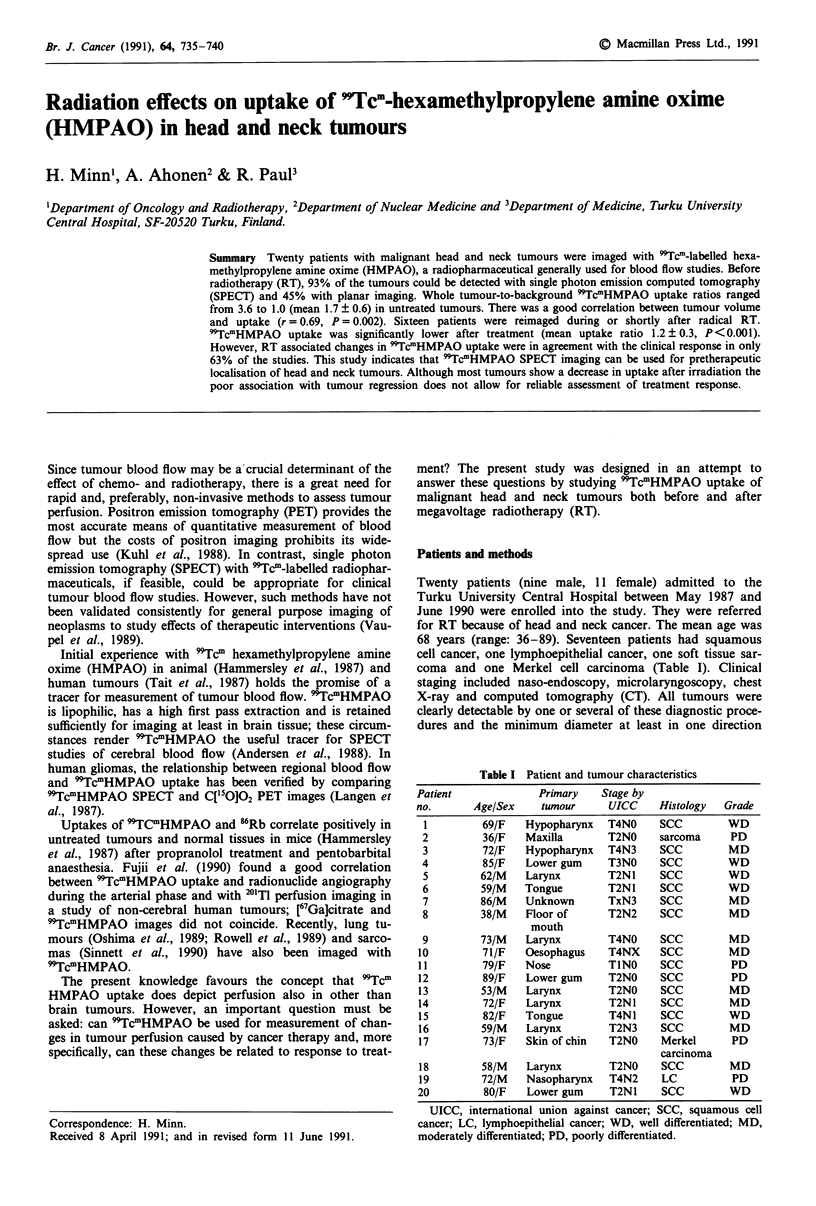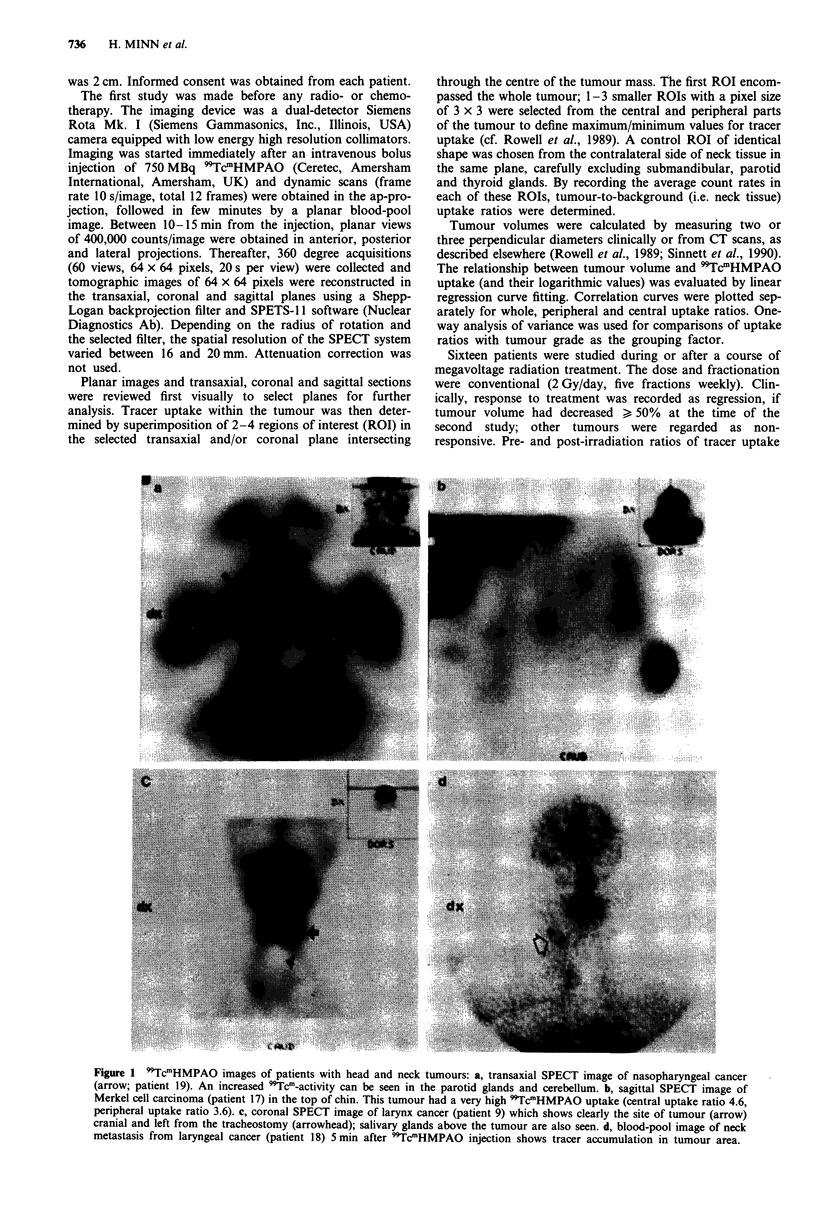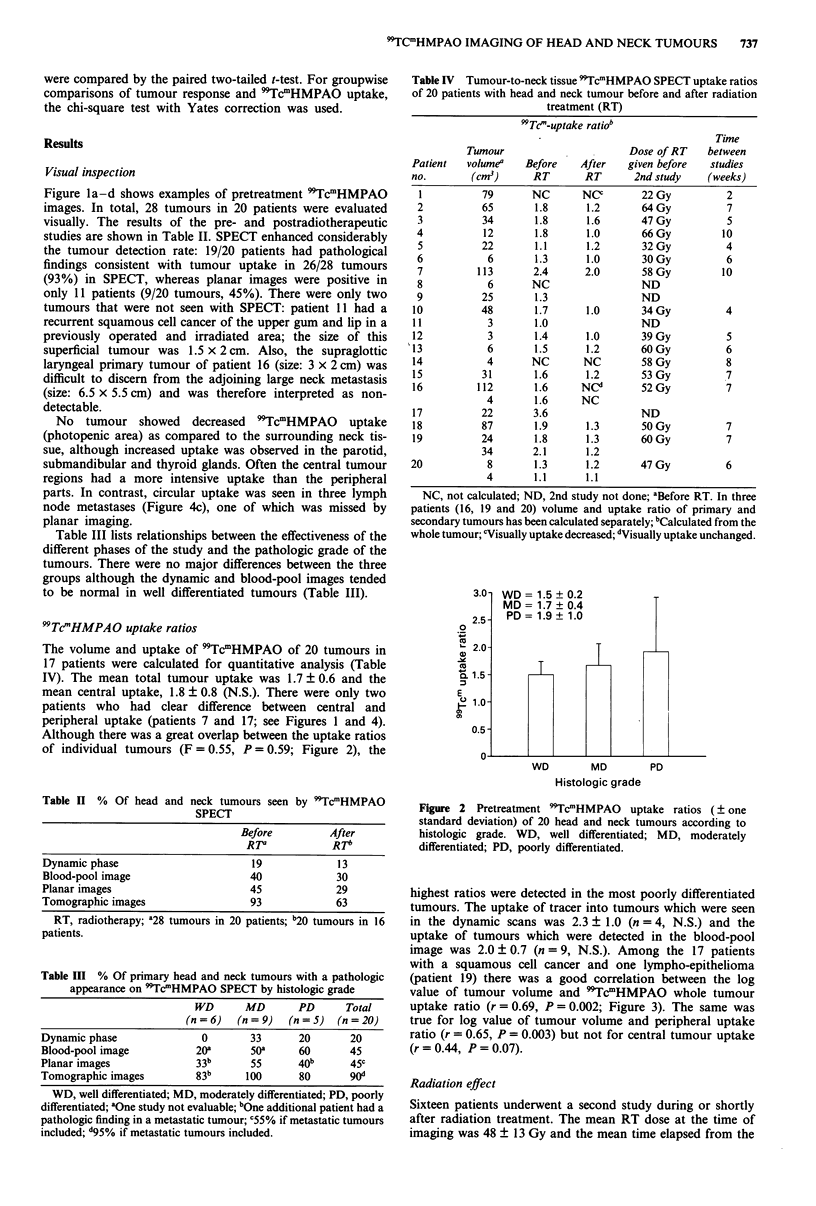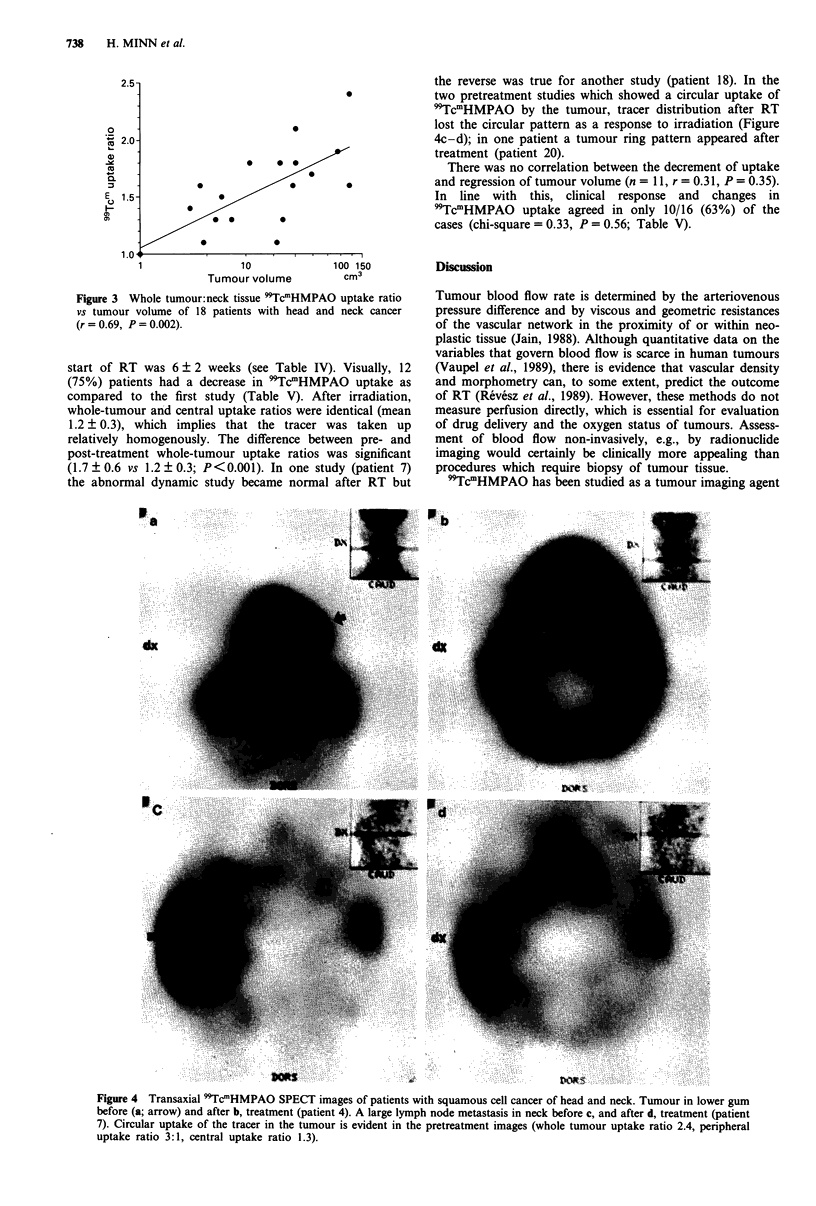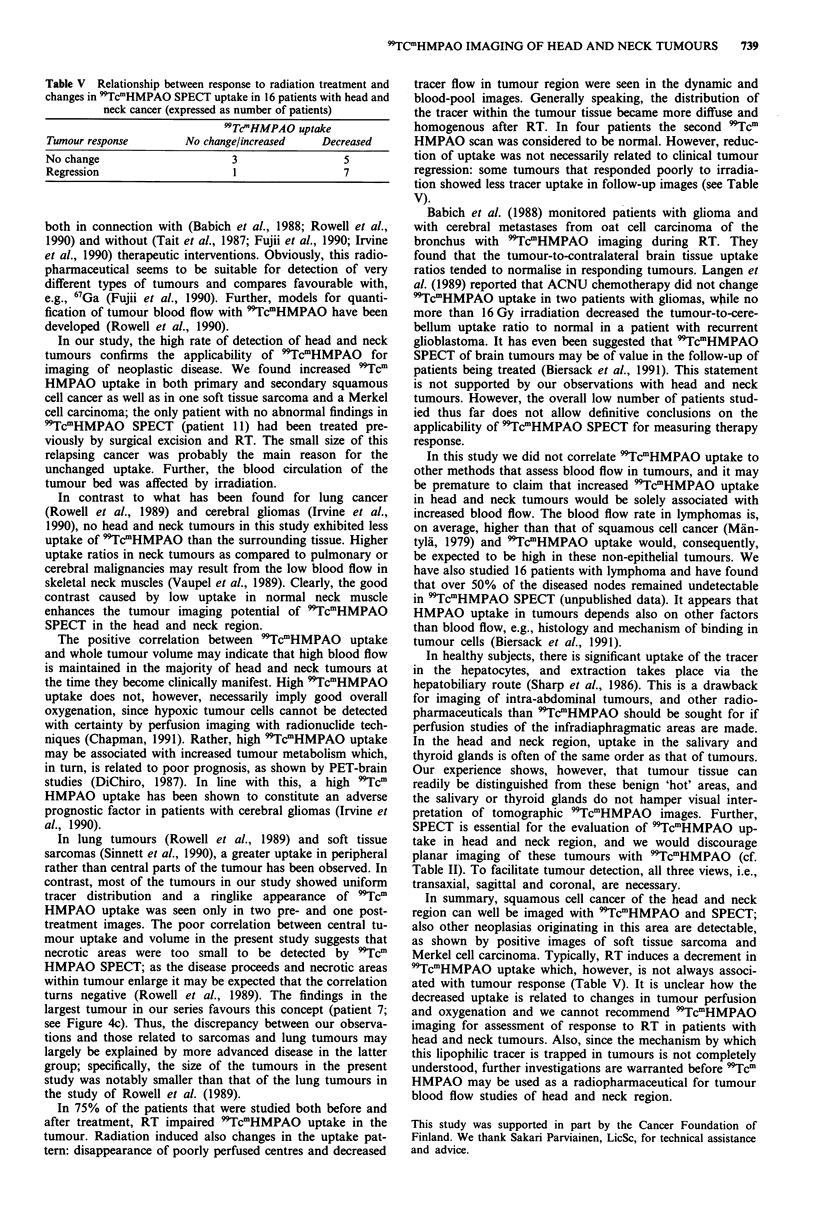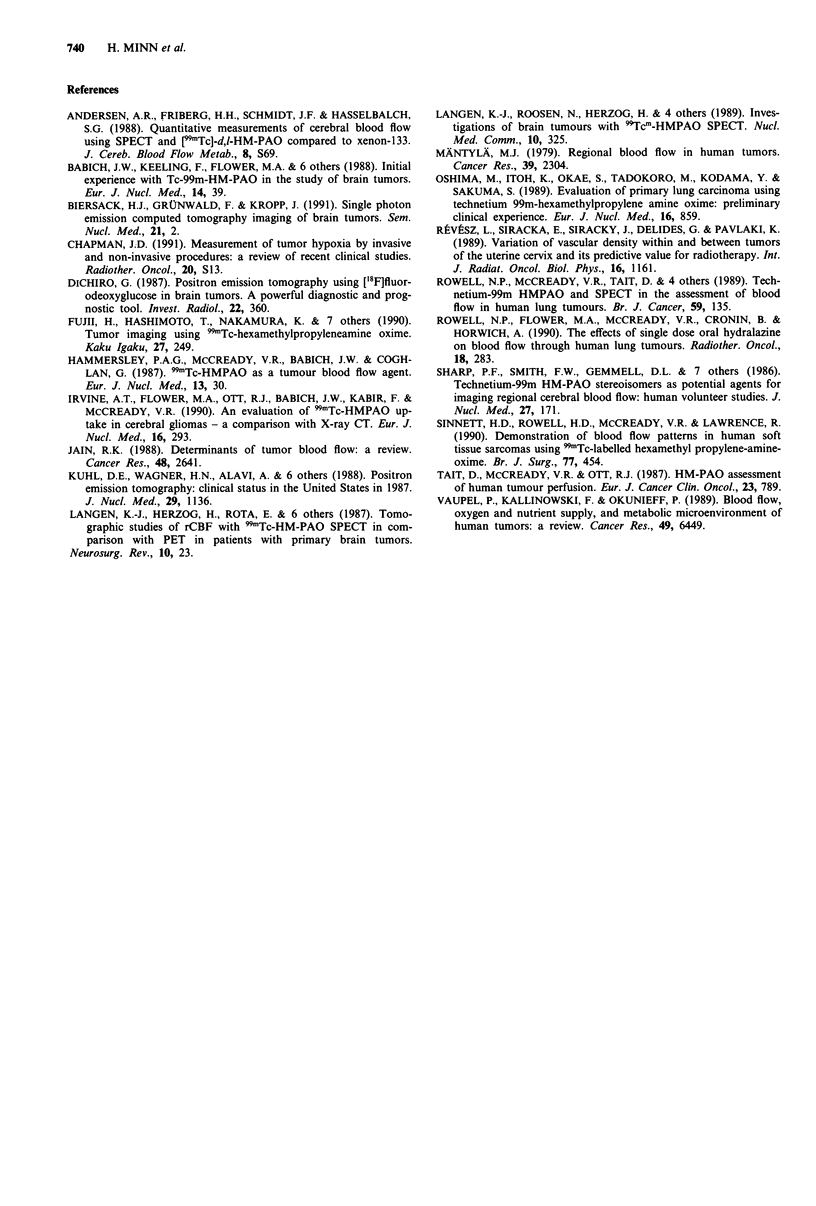# Radiation effects on uptake of 99Tcm-hexamethylpropylen amine oxime (HMPAO) in head and neck tumours.

**DOI:** 10.1038/bjc.1991.389

**Published:** 1991-10

**Authors:** H. Minn, A. Ahonen, R. Paul

**Affiliations:** Department of Oncology and Radiotherapy, Turku University Central Hospital, Finland.

## Abstract

**Images:**


Br. J. Cancer (1991), 64, 735-740 © Macmillan Press Ltd., 1991

### Radiation effects on uptake of $^{99}Tc^{m}$-hexamethylpropylene amine oxime (HMPAO) in head and neck tumours

H. Minn[1], A. Ahonen[2] & R. Paul[3]

[1]Department of Oncology and Radiotherapy, [2]Department of Nuclear Medicine and [3]Department of Medicine, Turku University Central Hospital, SF-20520 Turku, Finland.

**Summary** Twenty patients with malignant head and neck tumours were imaged with $^{99}Tc^{m}$-labelled hexamethylpropylene amine oxime (HMPAO), a radiopharmaceutical generally used for blood flow studies. Before radiotherapy (RT), 93% of the tumours could be detected with single photon emission computed tomography (SPECT) and 45% with planar imaging. Whole tumour-to-background $^{99}Tc^{m}$HMPAO uptake ratios ranged from 3.6 to 1.0 (mean 1.7 ± 0.6) in untreated tumours. There was a good correlation between tumour volume and uptake ($r = 0.69$, $P = 0.002$). Sixteen patients were reimaged during or shortly after radical RT. $^{99}Tc^{m}$HMPAO uptake was significantly lower after treatment (mean uptake ratio 1.2 ± 0.3, $P < 0.001$). However, RT associated changes in $^{99}Tc^{m}$HMPAO uptake were in agreement with the clinical response in only 63% of the studies. This study indicates that $^{99}Tc^{m}$HMPAO SPECT imaging can be used for pretherapeutic localisation of head and neck tumours. Although most tumours show a decrease in uptake after irradiation the poor association with tumour regression does not allow for reliable assessment of treatment response.

Since tumour blood flow may be a crucial determinant of the effect of chemo- and radiotherapy, there is a great need for rapid and, preferably, non-invasive methods to assess tumour perfusion. Positron emission tomography (PET) provides the most accurate means of quantitative measurement of blood flow but the costs of positron imaging prohibits its widespread use (Kuhl *et al.*, 1988). In contrast, single photon emission tomography (SPECT) with $^{99}Tc^{m}$-labelled radiopharmaceuticals, if feasible, could be appropriate for clinical tumour blood flow studies. However, such methods have not been validated consistently for general purpose imaging of neoplasms to study effects of therapeutic interventions (Vaupel *et al.*, 1989).

Initial experience with $^{99}Tc^{m}$ hexamethylpropylene amine oxime (HMPAO) in animal (Hammersley *et al.*, 1987) and human tumours (Tait *et al.*, 1987) holds the promise of a tracer for measurement of tumour blood flow. $^{99}Tc^{m}$HMPAO is lipophilic, has a high first pass extraction and is retained sufficiently for imaging at least in brain tissue; these circumstances render $^{99}Tc^{m}$HMPAO the useful tracer for SPECT studies of cerebral blood flow (Andersen *et al.*, 1988). In human gliomas, the relationship between regional blood flow and $^{99}Tc^{m}$HMPAO uptake has been verified by comparing $^{99}Tc^{m}$HMPAO SPECT and $C[^{15}O]O_2$ PET images (Langen *et al.*, 1987).

Uptakes of $^{99}TC^{m}$HMPAO and $^{86}Rb$ correlate positively in untreated tumours and normal tissues in mice (Hammersley *et al.*, 1987) after propranolol treatment and pentobarbital anaesthesia. Fujii *et al.* (1990) found a good correlation between $^{99}Tc^{m}$HMPAO uptake and radionuclide angiography during the arterial phase and with $^{201}Tl$ perfusion imaging in a study of non-cerebral human tumours; [$^{67}Ga$]citrate and $^{99}Tc^{m}$HMPAO images did not coincide. Recently, lung tumours (Oshima *et al.*, 1989; Rowell *et al.*, 1989) and sarcomas (Sinnett *et al.*, 1990) have also been imaged with $^{99}Tc^{m}$HMPAO.

The present knowledge favours the concept that $^{99}Tc^{m}$ HMPAO uptake does depict perfusion also in other than brain tumours. However, an important question must be asked: can $^{99}Tc^{m}$HMPAO be used for measurement of changes in tumour perfusion caused by cancer therapy and, more specifically, can these changes be related to response to treatment? The present study was designed in an attempt to answer these questions by studying $^{99}Tc^{m}$HMPAO uptake of malignant head and neck tumours both before and after megavoltage radiotherapy (RT).

Correspondence: H. Minn.
Received 8 April 1991; and in revised form 11 June 1991.

### Patients and methods

Twenty patients (nine male, 11 female) admitted to the Turku University Central Hospital between May 1987 and June 1990 were enrolled into the study. They were referred for RT because of head and neck cancer. The mean age was 68 years (range: 36-89). Seventeen patients had squamous cell cancer, one lymphoepithelial cancer, one soft tissue sarcoma and one Merkel cell carcinoma (Table I). Clinical staging included naso-endoscopy, microlaryngoscopy, chest X-ray and computed tomography (CT). All tumours were clearly detectable by one or several of these diagnostic procedures and the minimum diameter at least in one direction

**Table I** Patient and tumour characteristics

| *Patient no.* | *Age/Sex* | *Primary tumour* | *Stage by UICC* | *Histology* | *Grade* |
|---|---|---|---|---|---|
| 1 | 69/F | Hypopharynx | T4N0 | SCC | WD |
| 2 | 36/F | Maxilla | T2N0 | sarcoma | PD |
| 3 | 72/F | Hypopharynx | T4N3 | SCC | MD |
| 4 | 85/F | Lower gum | T3N0 | SCC | WD |
| 5 | 62/M | Larynx | T2N1 | SCC | WD |
| 6 | 59/M | Tongue | T2N1 | SCC | WD |
| 7 | 86/M | Unknown | TxN3 | SCC | MD |
| 8 | 38/M | Floor of mouth | T2N2 | SCC | MD |
| 9 | 73/M | Larynx | T4N0 | SCC | MD |
| 10 | 71/F | Oesophagus | T4NX | SCC | MD |
| 11 | 79/F | Nose | T1N0 | SCC | PD |
| 12 | 89/F | Lower gum | T2N0 | SCC | PD |
| 13 | 53/M | Larynx | T2N0 | SCC | MD |
| 14 | 72/F | Larynx | T2N1 | SCC | MD |
| 15 | 82/F | Tongue | T4N1 | SCC | WD |
| 16 | 59/M | Larynx | T2N3 | SCC | MD |
| 17 | 73/F | Skin of chin | T2N0 | Merkel carcinoma | PD |
| 18 | 58/M | Larynx | T2N0 | SCC | MD |
| 19 | 72/M | Nasopharynx | T4N2 | LC | PD |
| 20 | 80/F | Lower gum | T2N1 | SCC | WD |

UICC, international union against cancer; SCC, squamous cell cancer; LC, lymphoepithelial cancer; WD, well differentiated; MD, moderately differentiated; PD, poorly differentiated.

was 2 cm. Informed consent was obtained from each patient.

The first study was made before any radio- or chemotherapy. The imaging device was a dual-detector Siemens Rota Mk. I (Siemens Gammasonics, Inc., Illinois, USA) camera equipped with low energy high resolution collimators. Imaging was started immediately after an intravenous bolus injection of 750 MBq $^{99}Tc^{m}$HMPAO (Ceretec, Amersham International, Amersham, UK) and dynamic scans (frame rate 10 s/image, total 12 frames) were obtained in the ap-projection, followed in few minutes by a planar blood-pool image. Between 10–15 min from the injection, planar views of 400,000 counts/image were obtained in anterior, posterior and lateral projections. Thereafter, 360 degree acquisitions (60 views, 64 × 64 pixels, 20 s per view) were collected and tomographic images of 64 × 64 pixels were reconstructed in the transaxial, coronal and sagittal planes using a Shepp-Logan backprojection filter and SPETS-11 software (Nuclear Diagnostics Ab). Depending on the radius of rotation and the selected filter, the spatial resolution of the SPECT system varied between 16 and 20 mm. Attenuation correction was not used.

Planar images and transaxial, coronal and sagittal sections were reviewed first visually to select planes for further analysis. Tracer uptake within the tumour was then determined by superimposition of 2–4 regions of interest (ROI) in the selected transaxial and/or coronal plane intersecting through the centre of the tumour mass. The first ROI encompassed the whole tumour; 1–3 smaller ROIs with a pixel size of 3 × 3 were selected from the central and peripheral parts of the tumour to define maximum/minimum values for tracer uptake (cf. Rowell *et al.*, 1989). A control ROI of identical shape was chosen from the contralateral side of neck tissue in the same plane, carefully excluding submandibular, parotid and thyroid glands. By recording the average count rates in each of these ROIs, tumour-to-background (i.e. neck tissue) uptake ratios were determined.

Tumour volumes were calculated by measuring two or three perpendicular diameters clinically or from CT scans, as described elsewhere (Rowell *et al.*, 1989; Sinnett *et al.*, 1990). The relationship between tumour volume and $^{99}Tc^{m}$HMPAO uptake (and their logarithmic values) was evaluated by linear regression curve fitting. Correlation curves were plotted separately for whole, peripheral and central uptake ratios. One-way analysis of variance was used for comparisons of uptake ratios with tumour grade as the grouping factor.

Sixteen patients were studied during or after a course of megavoltage radiation treatment. The dose and fractionation were conventional (2 Gy/day, five fractions weekly). Clinically, response to treatment was recorded as regression, if tumour volume had decreased $\geq 50\%$ at the time of the second study; other tumours were regarded as non-responsive. Pre- and post-irradiation ratios of tracer uptake

**Figure 1** $^{99}Tc^{m}$HMPAO images of patients with head and neck tumours: **a**, transaxial SPECT image of nasopharyngeal cancer (arrow; patient 19). An increased $^{99}Tc^{m}$-activity can be seen in the parotid glands and cerebellum. **b**, sagittal SPECT image of Merkel cell carcinoma (patient 17) in the top of chin. This tumour had a very high $^{99}Tc^{m}$HMPAO uptake (central uptake ratio 4.6, peripheral uptake ratio 3.6). **c**, coronal SPECT image of larynx cancer (patient 9) which shows clearly the site of tumour (arrow) cranial and left from the tracheostomy (arrowhead); salivary glands above the tumour are also seen. **d**, blood-pool image of neck metastasis from laryngeal cancer (patient 18) 5 min after $^{99}Tc^{m}$HMPAO injection shows tracer accumulation in tumour area.

were compared by the paired two-tailed *t*-test. For groupwise comparisons of tumour response and $^{99}$Tc$^{m}$HMPAO uptake, the chi-square test with Yates correction was used.

### Results

### *Visual inspection*

Figure 1a–d shows examples of pretreatment $^{99}$Tc$^{m}$HMPAO images. In total, 28 tumours in 20 patients were evaluated visually. The results of the pre- and postradiotherapeutic studies are shown in Table II. SPECT enhanced considerably the tumour detection rate: 19/20 patients had pathological findings consistent with tumour uptake in 26/28 tumours (93%) in SPECT, whereas planar images were positive in only 11 patients (9/20 tumours, 45%). There were only two tumours that were not seen with SPECT: patient 11 had a recurrent squamous cell cancer of the upper gum and lip in a previously operated and irradiated area; the size of this superficial tumour was 1.5 × 2 cm. Also, the supraglottic laryngeal primary tumour of patient 16 (size: 3 × 2 cm) was difficult to discern from the adjoining large neck metastasis (size: 6.5 × 5.5 cm) and was therefore interpreted as non-detectable.

No tumour showed decreased $^{99}$Tc$^{m}$HMPAO uptake (photopenic area) as compared to the surrounding neck tissue, although increased uptake was observed in the parotid, submandibular and thyroid glands. Often the central tumour regions had a more intensive uptake than the peripheral parts. In contrast, circular uptake was seen in three lymph node metastases (Figure 4c), one of which was missed by planar imaging.

Table III lists relationships between the effectiveness of the different phases of the study and the pathologic grade of the tumours. There were no major differences between the three groups although the dynamic and blood-pool images tended to be normal in well differentiated tumours (Table III).

### *$^{99}$Tc$^{m}$HMPAO uptake ratios*

The volume and uptake of $^{99}$Tc$^{m}$HMPAO of 20 tumours in 17 patients were calculated for quantitative analysis (Table IV). The mean total tumour uptake was 1.7 ± 0.6 and the mean central uptake, 1.8 ± 0.8 (N.S.). There were only two patients who had clear difference between central and peripheral uptake (patients 7 and 17; see Figures 1 and 4). Although there was a great overlap between the uptake ratios of individual tumours ($F = 0.55$, $P = 0.59$; Figure 2), the highest ratios were detected in the most poorly differentiated tumours. The uptake of tracer into tumours which were seen in the dynamic scans was 2.3 ± 1.0 ($n = 4$, N.S.) and the uptake of tumours which were detected in the blood-pool image was 2.0 ± 0.7 ($n = 9$, N.S.). Among the 17 patients with a squamous cell cancer and one lympho-epithelioma (patient 19) there was a good correlation between the log value of tumour volume and $^{99}$Tc$^{m}$HMPAO whole tumour uptake ratio ($r = 0.69$, $P = 0.002$; Figure 3). The same was true for log value of tumour volume and peripheral uptake ratio ($r = 0.65$, $P = 0.003$) but not for central tumour uptake ($r = 0.44$, $P = 0.07$).

**Table II** % Of head and neck tumours seen by $^{99}$Tc$^{m}$HMPAO SPECT

| | *Before RT*[a] | *After RT*[b] |
|---|---|---|
| Dynamic phase | 19 | 13 |
| Blood-pool image | 40 | 30 |
| Planar images | 45 | 29 |
| Tomographic images | 93 | 63 |

RT, radiotherapy; [a]28 tumours in 20 patients; [b]20 tumours in 16 patients.

**Table III** % Of primary head and neck tumours with a pathologic appearance on $^{99}$Tc$^{m}$HMPAO SPECT by histologic grade

| | *WD (n = 6)* | *MD (n = 9)* | *PD (n = 5)* | *Total (n = 20)* |
|---|---|---|---|---|
| Dynamic phase | 0 | 33 | 20 | 20 |
| Blood-pool image | 20[a] | 50[a] | 60 | 45 |
| Planar images | 33[b] | 55 | 40[b] | 45[c] |
| Tomographic images | 83[b] | 100 | 80 | 90[d] |

WD, well differentiated; MD, moderately differentiated; PD, poorly differentiated; [a]One study not evaluable; [b]One additional patient had a pathologic finding in a metastatic tumour; [c]55% if metastatic tumours included; [d]95% if metastatic tumours included.

**Table IV** Tumour-to-neck tissue $^{99}$Tc$^{m}$HMPAO SPECT uptake ratios of 20 patients with head and neck tumour before and after radiation treatment (RT)

| *Patient no.* | *Tumour volume*[a] *(cm³)* | *$^{99}$Tc$^{m}$-uptake ratio*[b] *Before RT* | *After RT* | *Dose of RT given before 2nd study* | *Time between studies (weeks)* |
|---|---|---|---|---|---|
| 1 | 79 | NC | NC[c] | 22 Gy | 2 |
| 2 | 65 | 1.8 | 1.2 | 64 Gy | 7 |
| 3 | 34 | 1.8 | 1.6 | 47 Gy | 5 |
| 4 | 12 | 1.8 | 1.0 | 66 Gy | 10 |
| 5 | 22 | 1.1 | 1.2 | 32 Gy | 4 |
| 6 | 6 | 1.3 | 1.0 | 30 Gy | 6 |
| 7 | 113 | 2.4 | 2.0 | 58 Gy | 10 |
| 8 | 6 | NC | | ND | |
| 9 | 25 | 1.3 | | ND | |
| 10 | 48 | 1.7 | 1.0 | 34 Gy | 4 |
| 11 | 3 | 1.0 | | ND | |
| 12 | 3 | 1.4 | 1.0 | 39 Gy | 5 |
| 13 | 6 | 1.5 | 1.2 | 60 Gy | 6 |
| 14 | 4 | NC | NC | 58 Gy | 8 |
| 15 | 31 | 1.6 | 1.2 | 53 Gy | 7 |
| 16 | 112 | 1.6 | NC[d] | 52 Gy | 7 |
| | 4 | 1.6 | NC | | |
| 17 | 22 | 3.6 | | ND | |
| 18 | 87 | 1.9 | 1.3 | 50 Gy | 7 |
| 19 | 24 | 1.8 | 1.3 | 60 Gy | 7 |
| | 34 | 2.1 | 1.2 | | |
| 20 | 8 | 1.3 | 1.2 | 47 Gy | 6 |
| | 4 | 1.1 | 1.1 | | |

NC, not calculated; ND, 2nd study not done; [a]Before RT. In three patients (16, 19 and 20) volume and uptake ratio of primary and secondary tumours has been calculated separately; [b]Calculated from the whole tumour; [c]Visually uptake decreased; [d]Visually uptake unchanged.

**Figure 2** Pretreatment $^{99}$Tc$^{m}$HMPAO uptake ratios (± one standard deviation) of 20 head and neck tumours according to histologic grade. WD, well differentiated; MD, moderately differentiated; PD, poorly differentiated.

### *Radiation effect*

Sixteen patients underwent a second study during or shortly after radiation treatment. The mean RT dose at the time of imaging was 48 ± 13 Gy and the mean time elapsed from the

**Figure 3** Whole tumour:neck tissue $^{99}Tc^{m}$HMPAO uptake ratio *vs* tumour volume of 18 patients with head and neck cancer ($r = 0.69$, $P = 0.002$).

start of RT was 6 ± 2 weeks (see Table IV). Visually, 12 (75%) patients had a decrease in $^{99}Tc^{m}$HMPAO uptake as compared to the first study (Table V). After irradiation, whole-tumour and central uptake ratios were identical (mean 1.2 ± 0.3), which implies that the tracer was taken up relatively homogenously. The difference between pre- and post-treatment whole-tumour uptake ratios was significant (1.7 ± 0.6 *vs* 1.2 ± 0.3; $P < 0.001$). In one study (patient 7) the abnormal dynamic study became normal after RT but the reverse was true for another study (patient 18). In the two pretreatment studies which showed a circular uptake of $^{99}Tc^{m}$HMPAO by the tumour, tracer distribution after RT lost the circular pattern as a response to irradiation (Figure 4c–d); in one patient a tumour ring pattern appeared after treatment (patient 20).

There was no correlation between the decrement of uptake and regression of tumour volume ($n = 11$, $r = 0.31$, $P = 0.35$). In line with this, clinical response and changes in $^{99}Tc^{m}$HMPAO uptake agreed in only 10/16 (63%) of the cases (chi-square = 0.33, $P = 0.56$; Table V).

### Discussion

Tumour blood flow rate is determined by the arteriovenous pressure difference and by viscous and geometric resistances of the vascular network in the proximity of or within neoplastic tissue (Jain, 1988). Although quantitative data on the variables that govern blood flow is scarce in human tumours (Vaupel *et al.*, 1989), there is evidence that vascular density and morphometry can, to some extent, predict the outcome of RT (Révész *et al.*, 1989). However, these methods do not measure perfusion directly, which is essential for evaluation of drug delivery and the oxygen status of tumours. Assessment of blood flow non-invasively, e.g., by radionuclide imaging would certainly be clinically more appealing than procedures which require biopsy of tumour tissue.

$^{99}Tc^{m}$HMPAO has been studied as a tumour imaging agent

**Figure 4** Transaxial $^{99}Tc^{m}$HMPAO SPECT images of patients with squamous cell cancer of head and neck. Tumour in lower gum before (**a**; arrow) and after **b**, treatment (patient 4). A large lymph node metastasis in neck before **c**, and after **d**, treatment (patient 7). Circular uptake of the tracer in the tumour is evident in the pretreatment images (whole tumour uptake ratio 2.4, peripheral uptake ratio 3:1, central uptake ratio 1.3).

**Table V** Relationship between response to radiation treatment and changes in $^{99}$Tc$^{m}$HMPAO SPECT uptake in 16 patients with head and neck cancer (expressed as number of patients)

| *Tumour response* | *$^{99}$Tc$^{m}$HMPAO uptake* No change/increased | Decreased |
|---|---|---|
| No change | 3 | 5 |
| Regression | 1 | 7 |

both in connection with (Babich *et al.*, 1988; Rowell *et al.*, 1990) and without (Tait *et al.*, 1987; Fujii *et al.*, 1990; Irvine *et al.*, 1990) therapeutic interventions. Obviously, this radiopharmaceutical seems to be suitable for detection of very different types of tumours and compares favourable with, e.g., $^{67}$Ga (Fujii *et al.*, 1990). Further, models for quantification of tumour blood flow with $^{99}$Tc$^{m}$HMPAO have been developed (Rowell *et al.*, 1990).

In our study, the high rate of detection of head and neck tumours confirms the applicability of $^{99}$Tc$^{m}$HMPAO for imaging of neoplastic disease. We found increased $^{99}$Tc$^{m}$ HMPAO uptake in both primary and secondary squamous cell cancer as well as in one soft tissue sarcoma and a Merkel cell carcinoma; the only patient with no abnormal findings in $^{99}$Tc$^{m}$HMPAO SPECT (patient 11) had been treated previously by surgical excision and RT. The small size of this relapsing cancer was probably the main reason for the unchanged uptake. Further, the blood circulation of the tumour bed was affected by irradiation.

In contrast to what has been found for lung cancer (Rowell *et al.*, 1989) and cerebral gliomas (Irvine *et al.*, 1990), no head and neck tumours in this study exhibited less uptake of $^{99}$Tc$^{m}$HMPAO than the surrounding tissue. Higher uptake ratios in neck tumours as compared to pulmonary or cerebral malignancies may result from the low blood flow in skeletal neck muscles (Vaupel *et al.*, 1989). Clearly, the good contrast caused by low uptake in normal neck muscle enhances the tumour imaging potential of $^{99}$Tc$^{m}$HMPAO SPECT in the head and neck region.

The positive correlation between $^{99}$Tc$^{m}$HMPAO uptake and whole tumour volume may indicate that high blood flow is maintained in the majority of head and neck tumours at the time they become clinically manifest. High $^{99}$Tc$^{m}$HMPAO uptake does not, however, necessarily imply good overall oxygenation, since hypoxic tumour cells cannot be detected with certainty by perfusion imaging with radionuclide techniques (Chapman, 1991). Rather, high $^{99}$Tc$^{m}$HMPAO uptake may be associated with increased tumour metabolism which, in turn, is related to poor prognosis, as shown by PET-brain studies (DiChiro, 1987). In line with this, a high $^{99}$Tc$^{m}$ HMPAO uptake has been shown to constitute an adverse prognostic factor in patients with cerebral gliomas (Irvine *et al.*, 1990).

In lung tumours (Rowell *et al.*, 1989) and soft tissue sarcomas (Sinnett *et al.*, 1990), a greater uptake in peripheral rather than central parts of the tumour has been observed. In contrast, most of the tumours in our study showed uniform tracer distribution and a ringlike appearance of $^{99}$Tc$^{m}$ HMPAO uptake was seen only in two pre- and one post-treatment images. The poor correlation between central tumour uptake and volume in the present study suggests that necrotic areas were too small to be detected by $^{99}$Tc$^{m}$ HMPAO SPECT; as the disease proceeds and necrotic areas within tumour enlarge it may be expected that the correlation turns negative (Rowell *et al.*, 1989). The findings in the largest tumour in our series favours this concept (patient 7; see Figure 4c). Thus, the discrepancy between our observations and those related to sarcomas and lung tumours may largely be explained by more advanced disease in the latter group; specifically, the size of the tumours in the present study was notably smaller than that of the lung tumours in the study of Rowell *et al.* (1989).

In 75% of the patients that were studied both before and after treatment, RT impaired $^{99}$Tc$^{m}$HMPAO uptake in the tumour. Radiation induced also changes in the uptake pattern: disappearance of poorly perfused centres and decreased tracer flow in tumour region were seen in the dynamic and blood-pool images. Generally speaking, the distribution of the tracer within the tumour tissue became more diffuse and homogenous after RT. In four patients the second $^{99}$Tc$^{m}$ HMPAO scan was considered to be normal. However, reduction of uptake was not necessarily related to clinical tumour regression: some tumours that responded poorly to irradiation showed less tracer uptake in follow-up images (see Table V).

Babich *et al.* (1988) monitored patients with glioma and with cerebral metastases from oat cell carcinoma of the bronchus with $^{99}$Tc$^{m}$HMPAO imaging during RT. They found that the tumour-to-contralateral brain tissue uptake ratios tended to normalise in responding tumours. Langen *et al.* (1989) reported that ACNU chemotherapy did not change $^{99}$Tc$^{m}$HMPAO uptake in two patients with gliomas, while no more than 16 Gy irradiation decreased the tumour-to-cerebellum uptake ratio to normal in a patient with recurrent glioblastoma. It has even been suggested that $^{99}$Tc$^{m}$HMPAO SPECT of brain tumours may be of value in the follow-up of patients being treated (Biersack *et al.*, 1991). This statement is not supported by our observations with head and neck tumours. However, the overall low number of patients studied thus far does not allow definitive conclusions on the applicability of $^{99}$Tc$^{m}$HMPAO SPECT for measuring therapy response.

In this study we did not correlate $^{99}$Tc$^{m}$HMPAO uptake to other methods that assess blood flow in tumours, and it may be premature to claim that increased $^{99}$Tc$^{m}$HMPAO uptake in head and neck tumours would be solely associated with increased blood flow. The blood flow rate in lymphomas is, on average, higher than that of squamous cell cancer (Mäntylä, 1979) and $^{99}$Tc$^{m}$HMPAO uptake would, consequently, be expected to be high in these non-epithelial tumours. We have also studied 16 patients with lymphoma and have found that over 50% of the diseased nodes remained undetectable in $^{99}$Tc$^{m}$HMPAO SPECT (unpublished data). It appears that HMPAO uptake in tumours depends also on other factors than blood flow, e.g., histology and mechanism of binding in tumour cells (Biersack *et al.*, 1991).

In healthy subjects, there is significant uptake of the tracer in the hepatocytes, and extraction takes place via the hepatobiliary route (Sharp *et al.*, 1986). This is a drawback for imaging of intra-abdominal tumours, and other radiopharmaceuticals than $^{99}$Tc$^{m}$HMPAO should be sought for if perfusion studies of the infradiaphragmatic areas are made. In the head and neck region, uptake in the salivary and thyroid glands is often of the same order as that of tumours. Our experience shows, however, that tumour tissue can readily be distinguished from these benign 'hot' areas, and the salivary or thyroid glands do not hamper visual interpretation of tomographic $^{99}$Tc$^{m}$HMPAO images. Further, SPECT is essential for the evaluation of $^{99}$Tc$^{m}$HMPAO uptake in head and neck region, and we would discourage planar imaging of these tumours with $^{99}$Tc$^{m}$HMPAO (cf. Table II). To facilitate tumour detection, all three views, i.e., transaxial, sagittal and coronal, are necessary.

In summary, squamous cell cancer of the head and neck region can well be imaged with $^{99}$Tc$^{m}$HMPAO and SPECT; also other neoplasias originating in this area are detectable, as shown by positive images of soft tissue sarcoma and Merkel cell carcinoma. Typically, RT induces a decrement in $^{99}$Tc$^{m}$HMPAO uptake which, however, is not always associated with tumour response (Table V). It is unclear how the decreased uptake is related to changes in tumour perfusion and oxygenation and we cannot recommend $^{99}$Tc$^{m}$HMPAO imaging for assessment of response to RT in patients with head and neck tumours. Also, since the mechanism by which this lipophilic tracer is trapped in tumours is not completely understood, further investigations are warranted before $^{99}$Tc$^{m}$ HMPAO may be used as a radiopharmaceutical for tumour blood flow studies of head and neck region.

This study was supported in part by the Cancer Foundation of Finland. We thank Sakari Parviainen, LicSc, for technical assistance and advice.

### References

ANDERSEN, A.R., FRIBERG, H.H., SCHMIDT, J.F. & HASSELBALCH, S.G. (1988). Quantitative measurements of cerebral blood flow using SPECT and [$^{99m}$Tc]-*d,l*-HM-PAO compared to xenon-133. *J. Cereb. Blood Flow Metab.*, **8**, S69.

BABICH, J.W., KEELING, F., FLOWER, M.A. & 6 others (1988). Initial experience with Tc-99m-HM-PAO in the study of brain tumors. *Eur. J. Nucl. Med.*, **14**, 39.

BIERSACK, H.J., GRÜNWALD, F. & KROPP, J. (1991). Single photon emission computed tomography imaging of brain tumors. *Sem. Nucl. Med.*, **21**, 2.

CHAPMAN, J.D. (1991). Measurement of tumor hypoxia by invasive and non-invasive procedures: a review of recent clinical studies. *Radiother. Oncol.*, **20**, S13.

DICHIRO, G. (1987). Positron emission tomography using [$^{18}$F]fluorodeoxyglucose in brain tumors. A powerful diagnostic and prognostic tool. *Invest. Radiol.*, **22**, 360.

FUJII, H., HASHIMOTO, T., NAKAMURA, K. & 7 others (1990). Tumor imaging using $^{99m}$Tc-hexamethylpropyleneamine oxime. *Kaku Igaku*, **27**, 249.

HAMMERSLEY, P.A.G., MCCREADY, V.R., BABICH, J.W. & COGHLAN, G. (1987). $^{99m}$Tc-HMPAO as a tumour blood flow agent. *Eur. J. Nucl. Med.*, **13**, 30.

IRVINE, A.T., FLOWER, M.A., OTT, R.J., BABICH, J.W., KABIR, F. & MCCREADY, V.R. (1990). An evaluation of $^{99m}$Tc-HMPAO uptake in cerebral gliomas – a comparison with X-ray CT. *Eur. J. Nucl. Med.*, **16**, 293.

JAIN, R.K. (1988). Determinants of tumor blood flow: a review. *Cancer Res.*, **48**, 2641.

KUHL, D.E., WAGNER, H.N., ALAVI, A. & 6 others (1988). Positron emission tomography: clinical status in the United States in 1987. *J. Nucl. Med.*, **29**, 1136.

LANGEN, K.-J., HERZOG, H., ROTA, E. & 6 others (1987). Tomographic studies of rCBF with $^{99m}$Tc-HM-PAO SPECT in comparison with PET in patients with primary brain tumors. *Neurosurg. Rev.*, **10**, 23.

LANGEN, K.-J., ROOSEN, N., HERZOG, H. & 4 others (1989). Investigations of brain tumours with $^{99}$Tc$^{m}$-HMPAO SPECT. *Nucl. Med. Comm.*, **10**, 325.

MÄNTYLÄ, M.J. (1979). Regional blood flow in human tumors. *Cancer Res.*, **39**, 2304.

OSHIMA, M., ITOH, K., OKAE, S., TADOKORO, M., KODAMA, Y. & SAKUMA, S. (1989). Evaluation of primary lung carcinoma using technetium 99m-hexamethylpropylene amine oxime: preliminary clinical experience. *Eur. J. Nucl. Med.*, **16**, 859.

RÉVÉSZ, L., SIRACKA, E., SIRACKY, J., DELIDES, G. & PAVLAKI, K. (1989). Variation of vascular density within and between tumors of the uterine cervix and its predictive value for radiotherapy. *Int. J. Radiat. Oncol. Biol. Phys.*, **16**, 1161.

ROWELL, N.P., MCCREADY, V.R., TAIT, D. & 4 others (1989). Technetium-99m HMPAO and SPECT in the assessment of blood flow in human lung tumours. *Br. J. Cancer*, **59**, 135.

ROWELL, N.P., FLOWER, M.A., MCCREADY, V.R., CRONIN, B. & HORWICH, A. (1990). The effects of single dose oral hydralazine on blood flow through human lung tumours. *Radiother. Oncol.*, **18**, 283.

SHARP, P.F., SMITH, F.W., GEMMELL, D.L. & 7 others (1986). Technetium-99m HM-PAO stereoisomers as potential agents for imaging regional cerebral blood flow: human volunteer studies. *J. Nucl. Med.*, **27**, 171.

SINNETT, H.D., ROWELL, H.D., MCCREADY, V.R. & LAWRENCE, R. (1990). Demonstration of blood flow patterns in human soft tissue sarcomas using $^{99m}$Tc-labelled hexamethyl propylene-amine-oxime. *Br. J. Surg.*, **77**, 454.

TAIT, D., MCCREADY, V.R. & OTT, R.J. (1987). HM-PAO assessment of human tumour perfusion. *Eur. J. Cancer Clin. Oncol.*, **23**, 789.

VAUPEL, P., KALLINOWSKI, F. & OKUNIEFF, P. (1989). Blood flow, oxygen and nutrient supply, and metabolic microenvironment of human tumors: a review. *Cancer Res.*, **49**, 6449.